# ‘Making the Implicit Explicit’: The Recommended Steps of a Caesarean Section, a Delphi Study Among South African Experts

**DOI:** 10.1155/ogi/6656341

**Published:** 2026-07-08

**Authors:** Liesl de Waard, Rozemiek Neline Hannelore Wessels, Anke Heitkamp, Thomas van den Akker, G. S. Gebhardt

**Affiliations:** ^1^ Department of Obstetrics and Gynaecology, Faculty of Medicine and Health Sciences, Stellenbosch University, Parow, South Africa, sun.ac.za; ^2^ Athena Institute, Faculty of Science, VU University, Amsterdam, Netherlands, vu.nl; ^3^ Department of Obstetrics and Gynaecology, Amsterdam University Medical Centre, Amsterdam, Netherlands, uva.nl; ^4^ Department of Obstetrics and Gynaecology, Leiden University Medical Centre, Leiden, Netherlands, unicamp.br

**Keywords:** caesarean delivery, caesarean section, caesarean section steps, caesarean section training, safe caesarean section

## Abstract

**Background and Aims:**

In low‐ and middle‐income countries, including South Africa, caesarean section (CS)–associated maternal complications, some of which are attributed to lack of skill and training, are of concern. The performing and teaching of CSs are not standardised in South Africa. This study aimed to identify the comprehensive steps, including those that are implicit, of a CS, according to South African experts.

**Methods:**

A modified three‐round Delphi survey method was used. The steps were divided into preoperative, intraoperative and postoperative. Invitations were sent to South African obstetric experts for voluntary participation. The aimed sample size was 15, and consensus was reached according to the confidence intervals (CIs) calculated from the Likert scale results. For the first two rounds, a seven‐point Likert scale was used. In round three, only steps that had not reached consensus yet were presented to the panel as essential, substeps or excluded.

**Results:**

Invitations were sent to 44 experts: 28 (64%) completed round one, 20 (45%) round two and 19 (43%) round three. Sixty‐six steps were identified after three rounds: 9 essential/14 substeps preoperatively, 14 essential/15 substeps intraoperatively and 5 essential/9 substeps postoperatively.

**Conclusion:**

South African obstetric experts recommended 66 steps for performing a CS. This comprehensive list could contribute to the standardisation and training of CSs, which may help increase the safety and quality of CSs, especially in low‐ and middle‐income settings where junior doctors perform most of these surgeries, often without supervision.

## 1. Introduction

Women in Africa have a 50‐times higher mortality rate associated with caesarean section (CS) than those from high‐income countries [1]. Postoperative complications occurred in 17% (1 in 6) of women who underwent a CS with a mortality rate of 0.5% of all CSs [1]. CSs are most commonly performed by nonspecialist physicians [1]. In South Africa, there has been a concerning number of maternal deaths from bleeding at CS, and the National Committee for Confidential Enquiries into Maternal Deaths (NCCEMD) identified lack of skill and training as a contributory factor [2]. In a 2022 survey, South African interns perceived their training as insufficient to perform CSs safely [3]. The African Peri‐Operative Research Group (APORG) looked into strategies to combat haemorrhage‐associated morbidity and mortality around CSs, and one of their recommendations was to enforce training standards for CSs [4].

Like many other countries, South Africa teaches CSs through apprenticeship without formal training. Dividing a complex task into multiple distinct steps may improve teaching and learning [5]. Creating a comprehensive list of exact steps may assist in arriving at a more systematic assessment and feedback as part of an objective structured assessment guide for workplace‐based competency assessment [6, 7]. We learn not only from what is explicitly taught but also through observation and remodelling, as described by Bandura’s social learning theory [8]. For this reason, ‘making the implicit explicit’ can help to improve learning by arriving at more structured observations, descriptions and remodelling [9].

The literature concerning CSs mainly focuses on the technical steps of the procedure and the evidence on different approaches to intraoperative techniques [10]. Evidence‐based techniques for CSs have been well described, where they are available [10, 11]. The CORONIS trial investigated CS techniques in a large multicentre trial, including a 3‐year follow‐up [12], and could not find any significant differences in outcomes among various techniques, including blunt versus sharp abdominal entry, internal versus exterior closure of the uterus, double‐ versus single‐layer uterine closure, different suture materials for uterine closure and peritoneal closure versus nonclosure [12, 13]. Since many of these techniques are considered equally safe, it is difficult to postulate what accounts for the complications observed in the South African setting and similar settings where a lack of skills and training contributes to morbidity and mortality. Several questions arise, among others, whether perhaps steps are taken that are implicit or intuitive but are not accounted for in the described techniques.

Expert opinion has been employed in other medical practice and education task list creations, including obstetrics. Such expert opinion has been shown to hold promise in identifying steps in outlet forceps birth, identifying interventions for reducing peripartum haemorrhage around CSs and providing expert consensus on a basic life support checklist [4, 14–16]. Consensus‐based expert opinion is a valuable tool when evidence is lacking because of the combination of the knowledge and experience of experts with specific knowledge and skills [17]. The Delphi method is a validated technique by which such collective knowledge and expertise are anonymously combined to reach consensus [18, 19]. This study used the Delphi method to determine the steps in performing a safe CS.

## 2. Methods

A modified Delphi method was used to obtain consensus from a panel of South African obstetric experts [20]. The study protocol was approved by the Health Research and Ethics Committee of Stellenbosch University (N22/07/081) in June 2022, and the Western Cape Department of Health granted institutional permission. Participation was voluntary, and informed consent was obtained before participation. Participants were given the option of remaining anonymous or adding their details at the end of the survey for acknowledgement. Their details were not shared with other participants or considered during data analysis. The rating and input in the surveys remained anonymous. The Guidelines for Conducting and Reporting of Delphi Studies (CREDES) were used for the reporting of this study. The checklist is available as additional Table 1 [21].

### 2.1. Setting

This study was conducted in South Africa, where healthcare is provided in both the public and private sectors. The public sector provides healthcare to approximately 71% of the country’s population of more than 60 million [22]. Just over 1,000,000 births occur in the public sector annually, with a 28.8% CS rate, amounting to just under 300,000 CSs performed annually [23]. Most of these procedures are performed by medical officers (nonspecialist medical practitioners), as in most parts of Africa [1]. Training in South Africa is provided in public sector hospitals. For CSs, training starts during the first postgraduate year (compulsory internship) and occurs mainly through apprenticeship without any structured training programmes or assessments by seniors [24]. Medical interns are required by a health professional’s body to complete 10 supervised CSs during their internship rotation before performing the procedure independently [25].

### 2.2. Participants

The panel consisted of South African obstetric experts who were at the time of the study or had previously been actively involved in the clinical training of CSs in the public sector. The investigating team identified experts who were specialist obstetricians involved in CS training in the public sector. Invitations were sent through the various academic departments involved in specialist training in obstetrics and gynaecology in South Africa. Panellists were recruited through email invitations. Obstetricians employed at hospitals in different regions of South Africa and hospital settings were included to ensure that the study sample was diverse in experience, geographic and educational background, and type of hospital setting. The target sample size of this project was 15–30 respondents to increase the validity of the results. The minimum number needed for adequate results in a Delphi method is 10–15 [18].

### 2.3. Delphi Process

Members of the investigating team developed the first round of the survey. Qualtrics™ software was used for survey development, and a link was distributed through the recruitment emails. The response window was within 4 weeks, and a reminder email was sent after 1 week. The three rounds took place between 2 November 2022 and 23 May 2023. All participants were invited to participate in all three rounds, irrespective of whether they had completed the previous round.

**FIGURE 1:**
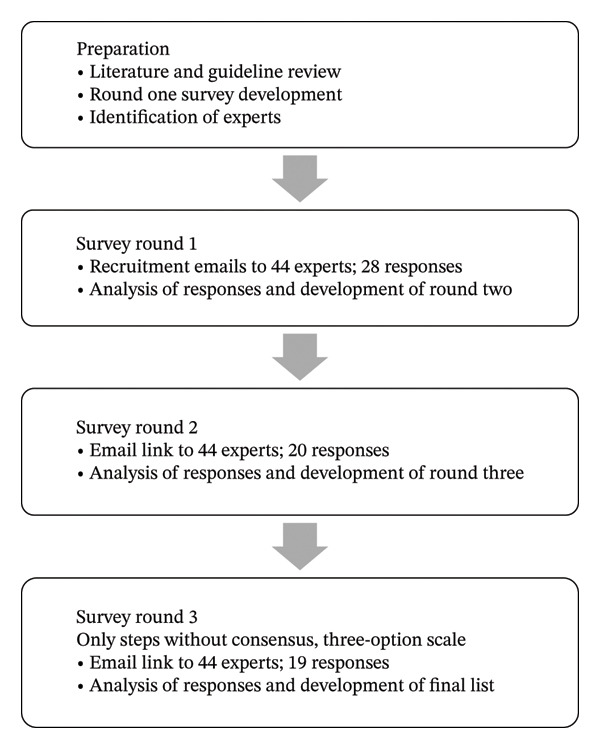
Research process flowchart.

In the first round, the participants received an email link to a survey in which steps were outlined that they could rate according to a seven‐point Likert scale. The purpose of the scale was to determine to what extent participants believed that the steps should be viewed as essential and distinct steps of a safe CS (*completely disagree*’—completely agree). After each phase (preoperative, intraoperative and postoperative), there was an option to suggest any additional steps. For the second round, the panel was presented with all the (newly) suggested steps from the previous round and was asked to rate them according to the seven‐point scale. All the steps of the previous round were shown with the mean and confidence interval (CI) scores. The steps that had already reached consensus were shown but were not for rating. For the third round, all the steps were presented, and those for which consensus had not been reached were rated according to three options as either an essential step (independent core steps for conducting a CS), a substep (steps that formed part of an essential step or that should be taken in certain clinical conditions depending on the context) or for exclusion. Figure 1 illustrates the research process (Figure 1).

### 2.4. Statistical Analysis

The data were analysed in SPSS 27. A seven‐point Likert scale, with *completely disagree* being one and *completely agree* being seven, was used in rounds one and two, and a three‐point scale was used in round three. Two‐tailed one‐sample *t*‐tests were performed to compute means and 95% CIs to determine whether an individual step had reached consensus [26, 27]. Cronbach’s alpha (*α*) was used to assess internal consistency and overall agreement across the final list of steps [26, 27]. The definition of consensus was set at a Cronbach’s alpha of ≥ 0.80, with an *α* of 0.80 or higher indicating good consensus and an *α* of 0.90 or higher indicating excellent consensus [26, 28]. Descriptive statistics were used to summarise participant characteristics and Delphi round responses. The definition of consensus based on means and CIs can be found in Table 1 below [27]. Responses to the open‐ended questions asking about potential additional steps were coded inductively.

**TABLE 1:** Definition of consensus based on 95% confidence intervals per survey round.

Interpretation	First round (1–7)	Second round (1–7)	Third round (1–3)
Unessential, exclude	Upper limit CI of < 4	Upper limit CI of < 4	Lower limit CI of < 1.7
Re‐evaluation in the following round	Lower limit CI of > 4 and < 6	Lower limit CI of > 4 and < 6	Not applicable
Substep	Not applicable	Not Applicable	Lower limit CI of > 1.7
Essential step	Lower limit CI of > 6	Lower limit CI of > 6	Lower limit CI of > 2.5

Abbreviation: CI, confidence interval.

## 3. Results

According to this three‐round Delphi survey, South African obstetric experts recommend 66 steps for performing a CS. These steps can be divided into 23 preoperative, 29 intraoperative and 14 postoperative steps, of which 28 can be classified as essential steps and 38 as recommended or substeps.

Twenty‐nine experts completed one or more rounds of the Delphi method. The panellists were affiliated with seven academic institutions and 14 hospitals, representing four provinces in South Africa. The majority, 17 (58.6%), were general specialist obstetricians, 7 (24.1%) had an additional PhD and 5 (17.2%) were subspecialists. The panel had extensive experience performing CSs; 27 (93%) had been performing CSs for more than 10 years, 24 (85.7%) were estimated to have performed more than 1000 CSs each, and 20 (68.9%) had more than 10 years of experience teaching CSs.

### 3.1. Delphi Round One

Using the modified Delphi method, round one consisted of a survey with steps that the expert panel could rate on their level of agreement, and there was an opportunity for the panel members to suggest additional steps. The initial steps are shown in Table 2.

**TABLE 2:** Results of Delphi round one.

	Caesarean section step	*N*	Mean	95% CI	Interpretation
*Preoperative phase*					
1	Decision‐making to perform CS	28	6.68	**[6.44–6.92]**	**Essential step**
2	Counselling and consent of the patient	28	6.71	**[6.54–6.89]**	**Essential step**
3	Preoperative risk assessment	28	6.86	**[6.68–7.03]**	**Essential step**

*Intraoperative phase*					
4	Skin incision	27	6.37	**[6.06–6.68]**	**Essential step**
5	Entry into the abdominal cavity	25	6.24	[5.86–6.62]	Re‐evaluation
6	Peritoneal opening	26	5.88	[5.29–6.48]	Re‐evaluation
7	Bladder reflection	26	5.77	[5.25–6.28]	Re‐evaluation
8	Uterine incision	25	6.52	**[6.23–6.81]**	**Essential step**
9	Amniotomy	26	5.54	[5.06–6.01]	Re‐evaluation
10	Foetal delivery	26	6.62	**[6.31–6.92]**	**Essential step**
11	Placental delivery	26	6.46	**[6.20–6.72]**	**Essential step**
12	Uterine closure	26	6.58	**[6.32–6.84]**	**Essential step**
13	Abdominal cavity closure	26	5.85	[5.27–6.43]	Re‐evaluation
14	Abdominal wall closure	26	6.42	**[6.14–6.71]**	**Essential step**

*Postoperative phase*					
15	Note‐making	28	6.89	**[6.77–7.01]**	**Essential step**
16	Nursing and postoperative instruction	28	6.68	**[6.40–6.96]**	**Essential step**
17	Postoperative clinical review	28	6.79	**[6.62–6.95]**	**Essential step**

*Note:* Essential steps are shown in bold.

Abbreviation: CI, confidence interval.

The panellists proposed an additional 20 preoperative, 20 intraoperative and 11 postoperative steps in round one. This list and the frequency with which the steps were mentioned can be found in additional Table 2. The first Delphi round reached good consensus with a Cronbach’s alpha of 0.85. All the proposed steps were included in the second round of the Delphi method to be evaluated for inclusion as distinct steps by all panellists.

### 3.2. Delphi Round Two

The second survey was completed by 20 (45.5%) out of the 44 contacted experts. Table 3 presents the CIs of round two. This Delphi round yielded a Cronbach’s alpha of 0.95, indicating excellent consensus. A total of eight steps, six in the preoperative phase and two in the intraoperative phase, had a 95% CI > 6, thus reaching consensus. These eight steps were added to the list of essential steps. All the steps reaching consensus were proposed as additional steps by experts in the previous round. Only three of the eight steps were proposed by more than one expert, while many additional steps were mentioned only once in the previous round. Many experts seemed to agree with the suggestions at least somewhat; neither of the steps had a 95% CI of < 4, meaning that no steps were deemed nonessential in this second survey round. The steps that did not reach consensus in this round were carried forward to round three of the Delphi process for re‐evaluation.

**TABLE 3:** Results of Delphi round two.

	Caesarean section step	*N*	Mean	95% CI	Interpretation
*Preoperative phase*					
1	Decision‐making to perform CS	*Essential step from round 1*			
2	Discuss decision with senior	20	5.30	[4.77–5.83]	Re‐evaluation
3	History and examination	20	6.30	[5.87–6.73]	Re‐evaluation
4	Counselling and consent of patient	*Essential step from round 1*			
5	Consent for blood transfusion	20	6.20	[5.68–6.20]	Re‐evaluation
6	Preoperative risk assessment	*Essential step from round 1*			
7	Assess the correct level of care/institution	20	6.25	[5.88–6.62]	Re‐evaluation
8	Inform anaesthetist	20	6.60	**[6.25–6.95]**	**Essential step**
9	Inform the theatre team	19	6.52	**[6.09–6.96]**	**Essential step**
10	Assess appropriate skill level of the team	20	6.15	[5.77–6.53]	Re‐evaluation
11	Assess haemoglobin level	20	6.60	**[6.32–6.88]**	**Essential step**
12	Assess appropriate theatre supplies and emergency blood	20	5.60	[5.02–6.18]	Re‐evaluation
13	Documentation of steps taken	20	6.20	[5.71–6.69]	Re‐evaluation
14	Preoperative medication and antibiotic prophylaxis	20	6.70	**[6.39–7.01]**	**Essential step**
15	Assess contraceptive choice	20	6.00	[5.57–6.43]	Re‐evaluation
16	Assess foetal heart presence in theatre	20	5.30	[4.55–6.05]	Re‐evaluation
17	Surgical planning	20	6.05	[5.58–6.52]	Re‐evaluation
18	Assess placental location	20	5.95	[5.51–6.39]	Re‐evaluation
19	Vaginal examination in theatre if in childbirth	20	5.20	[4.51–5.89]	Re‐evaluation
20	Insert an intravenous line and transurethral catheter	20	6.20	[5.68–6.72]	Re‐evaluation
21	Position in lateral tilt	20	5.90	[5.35–6.45]	Re‐evaluation
22	Infection prevention and control, skin and vaginal cleansing	20	6.45	**[6.01–6.89]**	**Essential step**
23	WHO surgical safety checklist	20	6.55	**[6.16–6.94]**	**Essential step**

*Intraoperative phase*					
24	Skin incision	*Essential step from round 1*			
25	Abdominal dissection	20	5.10	[4.36–5.84]	Re‐evaluation
26	Rectus sheath opening	20	5.30	[4.67–5.93]	Re‐evaluation
27	Peritoneal opening	20	5.15	[4.48–5.82]	Re‐evaluation
28	Safe adhesiolysis	20	5.35	[4.60–6.10]	Re‐evaluation
29	Bladder reflection	20	5.65	[5.10–6.20]	Re‐evaluation
30	Plan uterine entry before incision (presenting part, placenta previa/low or impacted head)	20	6.35	**[6.00–6.70]**	**Essential step**
31	Uterine incision	*Essential step from round 1*			
32	Amniotomy	19	4.73	[4.18–5.29]	Re‐evaluation
33	Foetal delivery	*Essential step from round 1*			
34	Rapid clamping of bleeders	20	5.15	[4.42–5.88]	Re‐evaluation
35	Delayed cord clamping	19	6.00	[5.57–6.43]	Re‐evaluation
36	Cord blood gas if indicated	19	5.57	[5.02–6.14]	Re‐evaluation
37	Oxytocin bolus	20	6.30	[5.92–6.68]	Re‐evaluation
38	Placental delivery	*Essential step from round 1*			
39	Send placenta for histology if indicated	18	5.44	[4.72–6.17]	Re‐evaluation
40	Compress lower uterine segment to limit bleeding	19	4.21	[3.50–4.92]	Re‐evaluation
41	Formally check if uterus is empty	20	5.55	[4.85–6.25]	Re‐evaluation
42	Check for lower segment uterine tears prior to suturing	20	5.65	[5.02–6.28]	Re‐evaluation
43	Consider exteriorisation of uterus if needed	20	5.25	[4.54–5.96]	Re‐evaluation
44	Uterine closure	*Essential step from round 1*			
45	Ensure uterus is contracted	20	6.15	[5.74–6.56]	Re‐evaluation
46	Ensure haemostasis	20	6.30	[5.96–6.64]	Re‐evaluation
47	Check visceral damage/bladder clear from sutures	20	5.95	[5.35–6.55]	Re‐evaluation
48	Inspection of abdominal cavity and examination of the uterus for injury	20	5.35	[4.67–6.03]	Re‐evaluation
49	Consider closure of the parietal peritoneum in special cases	19	4.57	[3.91–5.25]	Re‐evaluation
50	Check swabs and instruments	20	6.75	**[6.49–7.01]**	**Essential step**
51	Rectus sheath closure	20	5.50	[4.73–6.27]	Re‐evaluation
52	Approximation sutures if fat layer > 2 cm	19	5.73	[5.12–6.36]	Re‐evaluation
53	Skin closure	*Essential step from round 1*			
54	Wound dressing	18	5.55	[4.94–6.18]	Re‐evaluation

*Postoperative phase*					
55	Check uterus is well contracted	20	6.10	[5.58–6.62]	Re‐evaluation
56	Complete surgical safety checklist before leaving theatre	20	6.05	[5.54–6.56]	Re‐evaluation
57	Baby‐friendly steps, keep with mother, initiate breastfeeding	19	6.05	[5.51–6.60]	Re‐evaluation
58	Note‐making	*Essential step from round 1*			
59	Anaesthetic report	20	5.80	[5.26–6.34]	Re‐evaluation
60	Nursing and postoperative instruction	*Essential step from round 1*			
61	Monitor vital signs	20	5.85	[5.26–6.44]	Re‐evaluation
62	Monitor for signs of intra‐abdominal bleeding	20	5.95	[5.41–6.49]	Re‐evaluation
63	Postoperative risk assessment for thromboprophylaxis/antibiotics/postpartum haemorrhage	20	6.10	[5.62–6.58]	Re‐evaluation
64	Review analgesia	20	5.90	[5.42–6.38]	Re‐evaluation
65	Postoperative clinical review	*Essential step from round 1*			
66	Maternal mental health assessment	19	5.15	[4.51–5.81]	Re‐evaluation
67	Mobilise early	19	6.05	[5.61–6.49]	Re‐evaluation
68	Plan for subsequent childbirth	19	5.68	[5.23–6.14]	Re‐evaluation

*Note:* Essential steps are shown in bold.

Abbreviation: CI, confidence interval.

### 3.3. Delphi Round Three

The final survey round was completed by 19 (43.2%) of the 44 contacted experts, all of whom had already participated in at least one previous round. For the third‐round survey, the panel were asked to rate only the steps that had not reached consensus on a three‐point scale as either excluded, substep/recommended step or an essential step. The third survey round yielded a Cronbach’s alpha of 0.93. Eight additional steps had a lower 95% CI bound of > 2.5 and were deemed essential steps. Six were in the intraoperative phase, and two were in the postoperative phase. All 19 panellists rated ‘Ensure haemostasis’ as an essential step. While ‘Rectus sheath opening’ reached a relatively low CI score compared to other steps in round two (95% CI; 4.67, 5.93), it was one of the steps reaching a positive consensus in round three (95% CI; 2.52, 2.95). Two intraoperative steps (‘Compress lower uterine segment to limit bleeding’ and ‘Consider closure of the parietal peritoneum in special cases’) had a lower CI bound of < 1.7 and thus were considered nonessential and removed from the list entirely. The remaining steps were included in the list as recommended or substeps.

The results of this third round of voting by obstetric experts, shown in Table 4, comprise a final list containing 66 CS steps: 28 essential steps and 38 substeps. Twenty‐three steps were preoperative, 29 were intraoperative and 14 were postoperative.

**TABLE 4:** Results of Delphi round three.

	Caesarean section step	*N*	Mean	95% CI	Interpretation
*Preoperative phase*					
1	Decision‐making to perform CS	*Essential step from previous round*			
2	Discuss decision with senior	19	2.11	[1.75–2.46]	Substep
3	History and examination	19	2.74	[2.47–3.01]	Substep
4	Counselling and consent of patient	*Essential step from previous round*			
5	Consent for blood transfusion	19	2.74	[2.47–3.01]	Substep
6	Preoperative risk assessment	*Essential step from previous round*			
7	Assess the correct level of care/institution	19	2.47	[2.14–2.81]	Substep
8	Inform anaesthetist	*Essential step from previous round*			
9	Inform the theatre team	*Essential step from previous round*			
10	Assess appropriate skill level of the team	19	2.53	[2.23–2.82]	Substep
11	Assess haemoglobin level	*Essential step from previous round*			
12	Assess appropriate theatre supplies and emergency blood	19	2.42	[2.18–2.67]	Substep
13	Documentation of steps taken	19	2.47	[2.18–2.77]	Substep
14	Preoperative medication and antibiotic prophylaxis	*Essential step from previous round*			
15	Assess contraceptive choice	19	2.37	[2.08–2.66]	Substep
16	Assess foetal heart presence in theatre	19	2.42	[2.09–2.75]	Substep
17	Surgical planning	19	2.68	[2.45–2.91]	Substep
18	Assess placental location	19	2.63	[2.39–2.87]	Substep
19	Vaginal examination in theatre if in childbirth	19	2.47	[2.18–2.77]	Substep
20	Insert an intravenous line and transurethral catheter	19	2.74	[2.47–3.01]	Substep
21	Position in lateral tilt	19	2.68	[2.40–2.96]	Substep
22	Infection prevention and control, skin and vaginal cleansing	*Essential step from previous round*			
23	WHO surgical safety checklist	*Essential step from previous round*			

*Intraoperative phase*					
24	Skin incision	*Essential step from previous round*			
25	Abdominal dissection	19	2.37	[2.04–2.70]	Substep
26	Rectus sheath opening	19	2.74	**[2.52–2.95]**	**Essential step**
27	Peritoneal opening	19	2.68	[2.45–2.91]	Substep
28	Safe adhesiolysis	19	2.53	[2.23–2.82]	Substep
29	Bladder reflection	19	2.58	[2.33–2.82]	Substep
30	Plan uterine entry before incision (presenting part, placenta previa/low or impacted head)	*Essential step from previous round*			
31	Uterine incision	*Essential step from previous round*			
32	Amniotomy	19	2.05	[1.75–2.35]	Substep
33	Foetal delivery	*Essential step from previous round*			
34	Rapid clamping of bleeders	19	2.47	[2.14–2.81]	Substep
35	Delayed cord clamping	19	2.68	[2.45–2.91]	Substep
36	Cord blood gas if indicated	19	2.32	[2.04–2.60]	Substep
37	Oxytocin bolus	18	2.72	[2.44–3.01]	Substep
38	Placental delivery	*Essential step from previous round*			
39	Send placenta for histology if indicated	19	2.21	[1.91–2.51]	Substep
40	Compress lower uterine segment to limit bleeding	19	1.79	[*1.45–2.13*]	*Excluded*
41	Formally check if uterus is empty	19	2.74	**[2.52–2.95]**	**Essential step**
42	Check for lower segment uterine tears prior to suturing	19	2.84	**[2.66–3.02]**	**Essential step**
43	Consider exteriorisation of uterus if needed	19	2.21	[1.91–2.51]	Substep
44	Uterine closure	*Essential step from previous round*			
45	Ensure uterus is contracted	19	2.89	**[2.74–3.05]**	**Essential step**
46	Ensure haemostasis	19	3.00	**N/a**	**Essential step**
47	Check visceral damage/bladder clear from sutures	19	2.74	**[2.52–2.95]**	**Essential step**
48	Inspection of abdominal cavity and examination of the uterus for injury	19	2.42	[2.14–2.71]	Substep
49	Consider closure of the parietal peritoneum in special cases	19	1.79	[*1.45–2.13*]	*Excluded*
50	Check swabs and instruments	*Essential step from previous round*			
51	Rectus sheath closure	19	2.58	[2.25–2.91]	Substep
52	Approximation sutures if fat layer > 2 cm	19	2.53	[2.23–2.82]	Substep
53	Skin closure	*Essential step from previous round*			
54	Wound dressing	19	2.63	[2.34–2.92]	Substep

*Postoperative phase*					
55	Check uterus is well contracted	19	2.79	**[2.59–2.99]**	**Essential step**
56	Complete surgical safety checklist before leaving theatre	19	2.68	[2.36–3.01]	Substep
57	Baby‐friendly steps, keep with mother, initiate breastfeeding	19	2.63	[2.39–2.87]	Substep
58	Note‐making	*Essential step from previous round*			
59	Anaesthetic report	19	2.47	[2.10–2.85]	Substep
60	Nursing and postoperative instructions	*Essential step from previous round*			
61	Monitor vital signs	19	2.84	**[2.66–3.02]**	**Essential step**
62	Monitor for signs of intra‐abdominal bleeding	19	2.58	[2.29–2.87]	Substep
63	Postoperative risk assessment for thromboprophylaxis/antibiotics/postpartum haemorrhage	19	2.68	[2.45–2.91]	Substep
64	Review analgesia	19	2.63	[2.39–2.87]	Substep
65	Postoperative clinical review	*Essential step from previous round*			
66	Maternal mental health assessment	19	2.11	[1.79–2.42]	Substep
67	Mobilise early	19	2.63	[2.34–2.92]	Substep
68	Plan for subsequent childbirth	19	2.47	[2.18–2.77]	Substep

*Note:* Essential steps are shown in bold.

Abbreviation: CI, confidence interval.

The final comprehensive list of steps is shown in Table 5. A total of 66 steps were identified; 23 preoperative, 29 intraoperative and 14 postoperative, of which 28 are classified as essential steps and 38 as substeps. In the preoperative phase, there are 9 essential steps and 14 substeps, in the intraoperative phase, there are 14 essential steps and 15 substeps, and in the postoperative phase, there are 5 essential steps and 9 substeps.

**TABLE 5:** Final list of caesarean section steps.

Steps toward a safe caesarean section by South African experts (28 essential steps and 38 substeps)
*Preoperative phase* (9 essential steps and 14 substeps)
**1. Decision-making to perform caesarean section**
*2. Discuss decision with senior*
*3. History and examination*
**4. Counselling and consent of patient**
*5. Consent for blood transfusion*
**6. Preoperative risk assessment**
*7. Assess the correct level of care/institution*
**8. Inform anaesthetist**
**9. Inform the theatre team**
*10. Assess appropriate skill level of team*
**11. Assess haemoglobin level**
*12. Assess appropriate theatre supplies and emergency blood*
*13. Documentation of steps taken*
**14. Preoperative medication and antibiotic prophylaxis**
*15. Assess contraceptive choice*
*16. Assess foetal heart presence in theatre*
*17. Surgical planning*
*18. Assess placental location*
*19. Vaginal examination in theatre if in childbirth*
*20. Insert intravenous line and transurethral catheter*
*21. Position in lateral tilt*
**22. Infection prevention and control, skin and vaginal cleansing**
**23. WHO surgical safety checklist**

*Intraoperative phase* (14 essential steps and 15 substeps)
**24. Skin incision**
*25. Abdominal dissection*
**26. Rectus sheath opening**
*27. Peritoneal opening*
*28. Safe adhesiolysis*
*29. Bladder reflection*
**30. Plan uterine entry before incision (presenting part, placenta previa/low or impacted head)**
**31. Uterine incision**
*32. Amniotomy*
**33. Foetal delivery**
*34. Rapid clamping of bleeders*
*35. Delayed cord clamping*
*36. Cord blood gas if indicated*
*37. Oxytocin bolus*
**38. Placental delivery**
*39. Send placenta for histology if indicated*
**40. Formally check if uterus is empty**
**41. Check for lower segment uterine tears prior to suturing**
*42. Consider exteriorisation of uterus if needed*
**43. Uterine closure**
**44. Ensure uterus is contracted**
**45. Ensure haemostasis**
**46. Check visceral damage/bladder clear from sutures**
*47. Inspection of abdominal cavity and examination of the uterus for injury*
**48. Check swabs and instruments**
*49. Rectus sheath closure*
*50. Approximation sutures if fat layer > 2 cm*
**51. Skin closure**
*52. Wound dressing*

*Postoperative phase* (5 essential steps and 9 substeps)
**53. Check uterus is well contracted**
*54. Complete surgical safety checklist before leaving theatre*
*55. Baby friendly steps, keep with mother, initiate breastfeeding*
**56. Note-making**
*57. Anaesthetic report*
**58. Nursing and postoperative instruction**
**59. Monitor vital signs**
*60. Monitor for signs of intra-abdominal bleeding*
*61. Postoperative risk assessment for thromboprophylaxis/antibiotics/postpartum haemorrhage*
*62. Review analgesia*
**63. Postoperative clinical review**
*64. Maternal mental health assessment*
*65. Mobilise early*
*66. Plan for subsequent birth*

*Note*: Essential steps are in bold, and substeps are indented in italics.

## 4. Discussion

This modified Delphi study developed a comprehensive list of steps to perform a CS according to South African obstetric experts. To the best of our knowledge, this study is the first attempt to create a comprehensive list of essential CS steps to be applied in the South African context.

The Delphi method reached consensus on the essential steps for a safe CS. All three rounds of Cronbach’s alpha indicated good or excellent agreement. Most of the proposed steps were accepted in the final list as essential steps or recommended substeps. Only two steps, both intraoperative, were excluded from the final list of CS steps.

The International Federation of Gynaecology and Obstetrics (FIGO) recognized the concern regarding maternal morbidity and mortality associated with CSs and published best practice recommendations for surgical techniques to perform CSs in 2023 [29]. These recommendations are based on available evidence as well as best practice recommendations. The recommendations contain five points regarding preoperative steps, which include techniques and interventions for the prevention of surgical site infection such as skin and vaginal preparation as well as the use of drapes [29]. When comparing the preoperative steps identified in this study, steps regarding preoperative medication, skin and vaginal cleansing as well as the World Health Organization (WHO) safety checklist were all included. What the Delphi study adds in the preoperative phase that is not discussed in the FIGO recommendations are steps for decision‐making, informed consent and counselling, risk assessment and communication with the theatre team—which are standard of care and perhaps not considered techniques. For the intraoperative phase, there are 10 main points discussing details of type of skin incision, uterine incision approaches to foetal extraction, placenta and cord management, and abdominal and wound closure in the FIGO recommendations [29]. In the Delphi study, these steps were all mentioned. In addition, ‘Plan uterine entry before incision (presenting part, placenta previa/low or impacted head)’ was added as an essential step; again, not a technique but an important and sometimes implicit or intuitive step that experts take and that in junior or inexperienced hands should be done explicitly. The same goes for steps such as ‘Ensure the uterus is contracted’, ‘Ensure haemostasis’ and ‘Check for visceral damage’, steps that are carried out automatically by experts that could prevent or identify complications if performed explicitly by inexperienced surgeons. The FIGO recommendations summarise the available evidence on the technical steps [29].

This Delphi study provides a comprehensive list of technical, intuitive and nontechnical steps. This list of steps is from South African experts who work in the public sector, and the list could change considering the specific context. The use of the Delphi method in developing clinical guidelines for the management of placenta accreta spectrum disorders in Colombia highlighted the importance of adjusting international guidelines to content‐specific guidelines considering local resources [30].

Competency‐based medical education has been shown to be vital in teaching technical skills [7, 31]. Standardised checklists or task lists are becoming more common in research and education. An example of such is the Consensus‐based Checklist of Educational Resources on Adult Basic Life Support and Technical Competencies in Distal Radius Fracture Fixation, with a final list of 38 steps [32]. The aim of this study was similar to that of other studies: to inform education, assessment and research and to step toward standardising CS training [33].

The strengths of this study include the relatively large sample size of the Delphi tool compared to what is reportedly necessary to generate adequate results. The expert panel was rich in experience, well qualified and diverse. The online Delphi design maintained anonymity and avoided confrontation among participants or the status, seniority or perceived expertise, and it limited the risk of voting being influenced by these factors. The Delphi tool reached a high level of internal consistency (high Cronbach’s alpha) in all three rounds, indicating the reliability of the consensus.

Regarding limitations, the results may not be generalisable to other contexts because the research was designed to be context‐specific. However, this could also be considered a strength because the focus on applying the list of CS steps to South African practice means that the results fit the intended research setting well. The methods are thoroughly described to ensure the replicability of the study in other countries with contexts similar to South Africa. Another potential limitation of the research is sampling bias. Obstetricians and gynaecologists in the private sector, as well as midwives, were excluded from the expert panel as they were not involved in CS training, but could have added valuable insights. Additionally, 12 out of 29 Delphi panellists were affiliated with Stellenbosch University. This affiliation and their education may have influenced the panellists’ opinions, which may have slightly skewed the results. Furthermore, these participants likely had experienced similar training, which could be considered a limitation. Although they still represent diverse clinical settings and levels of expertise. Only 19 respondents completed the final round, with a 68% attrition rate, which should be further considered as a limitation. The list created has not been tested for use in either a clinical or teaching setting, and further studies should assess the validity in simulation and clinical training settings.

Further research recommendations from the team include assessing the applicability of the steps to real‐life situations. Stakeholder groups, such as midwives, nurses and junior doctors, should be involved in obtaining more well‐rounded and richer data. Repeating this process in other‐resourced countries or even on an international scale could improve the generalisability of the findings.

This Delphi process from South African experts achieved consensus on 28 essential steps and 38 substeps for performing a CS in South Africa. This can serve as a step toward standardizing the training and assessment of CSs in South Africa and other parts of the world. This comprehensive list aims to make intuitive and implicit steps explicit. This may ultimately lead to improved care and quality of CS births. It would be valuable if a training or assessment tool were designed based on these findings because it would be flexible, easily adaptable and reviewed within the context in which it is used.

## Author Contributions

All the authors contributed to the conceptualization of the project. Liesl de Waard: protocol development, data collection and management, and manuscript writing. Rozemiek Neline Hannelore Wessels: protocol development, data collection and management, data analysis, and manuscript writing. Anke Heitkamp: protocol development and manuscript writing and editing. Thomas van den Akker: protocol development and manuscript writing and editing. G. S. Gebhardt: protocol development and manuscript writing and editing.

## Funding

LDW received a PhD grant from the Discovery Foundation, which supports this work.

## Disclosure

A prior version of this manuscript is available as a preprint [34]. All authors have read and approved the final version of the manuscript. L de Waard has full access to all of the data in this study and takes complete responsibility for the integrity of the data and the accuracy of the data analysis.

L de Waard affirms that this manuscript is an honest, accurate and transparent account of the study being reported, that no important aspects of the study have been omitted, and that any discrepancies from the study as planned have been explained. The Discovery Foundation had no influence on the study design, data collection, interpretation or reporting of this research.

## Ethics Statement

The study protocol was approved by the health research and ethics committee of Stellenbosch University (N22/07/081) and the Western Cape Department of Health granted institutional permission.

## Consent

Participation was voluntary, and informed consent was obtained prior to participation. This included permission to publish the results of the study.

## Conflicts of Interest

The authors declare no conflicts of interest.

## Supporting Information

Additional supporting information can be found online in the Supporting Information section.

## Supporting information


**Supporting Information** Additional Table 1: Recommendations for the Conducting and Reporting of Delphi Studies (CREDES). This additional table outlines the CREDES recommendations and supporting page numbers. Additional Table 2: Additional Steps Proposed by Panellists in Round One. This table shows all additional proposed steps from the panellist in the first round.

## Data Availability

The dataset used and analysed during the study is available from the corresponding author on reasonable request.
