# Supplementary material for: ‘Making the Implicit Explicit’: The Recommended Steps of a Caesarean Section, a Delphi Study Among South African Experts
Source: Obstet Gynecol Int. 2026 Jul 8;2026:6656341. doi: 10.1155/ogi/6656341 (PMC13342840; doi:10.1155/ogi/6656341)
Supplement: Supplementary file 1 — Supporting Information Additional Table 1: Recommendations for the Conducting and Reporting of Delphi Studies (CREDES). This additional table outlines the CREDES recommendations and supporting page numbers. Additional Table 2: Additional Steps Proposed by Panellists in Round One. This table shows all additional proposed steps from the panellist in the first round. [file OGI-2026-6656341-s001.docx]

***Additional Table 1: Recommendations for the Conducting and Reporting of Delphi Studies (CREDES)***

| **Items for Reporting** | **Page reported on** |
| --- | --- |
| **8.** **Purpose and rationale.** The purpose of the study should be clearly defined and demonstrate the appropriateness of the use of the Delphi technique as a method to achieve the research aim. A rationale for the choice of the Delphi technique as the most  suitable method needs to be provided | 4 |
| **9. Expert panel.** Criteria for the selection of experts and transparent information on recruitment of the expert panel, sociodemographic details including information on expertise regarding the topic in question, (non)response and response rates  over the ongoing iterations should be reported | 5-6 |
| **10. Description of the methods.** The methods employed need to be comprehensible; this includes information on preparatory steps | 5-7 |
| **11. Procedure.** Flow chart to illustrate the stages of the Delphi process, including a preparatory phase, the actual ‘Delphi rounds’, interim steps of data processing and analysis, and concluding steps | 7 (Figure 1) |
| **12. Definition and attainment of consensus.** It needs to be comprehensible to the reader how consensus was achieved throughout the process, including strategies to deal with non-consensus | 8 (Table 7) |
| **13. Results.** Reporting of results for each round separately is highly advisable in order to make the evolving of consensus over the rounds transparent. This includes figures showing the average group response, changes between rounds, as well as any modifications of the survey instrument such as deletion, addition or modification of survey items based on previous rounds | 8-14 |
| **14. Discussion of limitations.** Reporting should include a critical reflection of potential limitations and their impact of the resulting guidance | 16 |
| **15. Adequacy of conclusions.** The conclusions should adequately reflect the outcomes of the Delphi study with a view to the scope and applicability of the resulting practice guidance | 17 |
| **16. Publication and dissemination.** | This article is submitted for publication. |

***Additional Table 2: Additional Steps Proposed by Panellists in Round One***

| Proposed step | Number of respondents |
| --- | --- |
| PREOPERATIVE PHASE |  |
| Discuss decision to perform caesarean section with senior | n = 1 |
| History and examination | n = 3 |
| Consent for blood transfusion | n = 1 |
| Assess the correct level of care/institution | n = 6 |
| Inform anaesthetist | n = 1 |
| Inform theatre team | n = 1 |
| Assess appropriate skill level of team | n = 5 |
| Assess haemoglobin level | n = 1 |
| Assess appropriate theatre supplies and emergency blood | n = 2 |
| Documentation of steps taken | n = 1 |
| Pre-operative medication and antibiotic prophylaxis | n = 5 |
| Assess contraceptive choice | n = 3 |
| Assess fetal heart presence in theatre | n = 2 |
| Surgical planning | n = 3 |
| Assess placental location | n = 1 |
| Vaginal examination in theatre if in childbirth | n = 2 |
| Insert intravenous line and transurethral catheter | n = 1 |
| Position in lateral tilt | n = 1 |
| Infection prevention and control, skin and vaginal cleansing | n = 3 |
| WHO surgical safety checklist | n = 3 |
|  |  |
| INTRAOPERATIVE PHASE |  |
| Abdominal dissection | n = 1 |
| Safe adhesiolysis | n = 1 |
| Plan uterine entry before incision (presenting part, placenta previa/low or impacted head) | n = 1 |
| Rapid clamping of bleeders | n = 1 |
| Delayed cord clamping | n = 1 |
| Cord blood gas if indicated | n = 2 |
| Oxytocin bolus | n = 2 |
| Send placenta for histology if indicated | n = 1 |
| Compress lower uterine segment to limit bleeding | n = 1 |
| Formally check if uterus is empty | n = 2 |
| Check for lower segment uterine tears prior to suturing | n = 1 |
| Consider exteriorization of uterus if needed | n = 2 |
| Ensure uterus is contracted | n = 2 |
| Ensure haemostasis | n = 2 |
| Ensure bladder clear from sutures | n = 1 |
| Inspection of abdominal cavity and examination of the uterus for injury | n = 2 |
| Consider closure of the parietal peritoneum in special cases | n = 2 |
| Check swabs and instruments | n = 1 |
| Approximation sutures if fat layer >2 cm | n = 1 |
| Wound dressing | n = 2 |
|  |  |
| POSTOPERATIVE PHASE |  |
| Check if uterus is well contracted | n = 4 |
| Complete surgical safety check list before leaving theatre | n = 2 |
| Baby-friendly steps, keep with mother, initiate breastfeeding | n = 2 |
| Anaesthetic report | n = 1 |
| Monitor vital signs | n = 6 |
| Monitor for signs of intra-abdominal bleeding | n = 6 |
| Postoperative risk assessment for thromboprophylaxis/antibiotics/postpartum haemorrhage | n = 4 |
| Review analgesia | n = 2 |
| Maternal mental health assessment | n = 1 |
| Mobilise early | n = 1 |
| Plan for subsequent childbirth | n = 1 |
